# Transformer-based artificial neural networks for the conversion between chemical notations

**DOI:** 10.1038/s41598-021-94082-y

**Published:** 2021-07-20

**Authors:** Lev Krasnov, Ivan Khokhlov, Maxim V. Fedorov, Sergey Sosnin

**Affiliations:** 1 Center for Computational and Data-Intensive Science and Engineering, Skolkovo Institute of Science and Technology , Bolshoy Boulevard 30, bld. 1, Moscow, 121205 Russia; 2 Syntelly LLC, Bolshoy Boulevard 30, bld. 1, Moscow, 121205 Russia; 3 Department of Chemistry, Lomonosov Moscow State University, GSP-1, 1-3 Leninskiye Gory, Moscow, 119991 Russia

**Keywords:** Cheminformatics, Information technology, Chemistry

## Abstract

We developed a Transformer-based artificial neural approach to translate between SMILES and IUPAC chemical notations: *Struct2IUPAC* and *IUPAC2Struct*. The overall performance level of our model is comparable to the rule-based solutions. We proved that the accuracy and speed of computations as well as the robustness of the model allow to use it in production. Our showcase demonstrates that a neural-based solution can facilitate rapid development keeping the required level of accuracy. We believe that our findings will inspire other developers to reduce development costs by replacing complex rule-based solutions with neural-based ones.

## Introduction

Before the Information Age, chemical names were a universal language for description of chemical structures. At the infancy stage of organic chemistry, there were no common rules for the naming of chemical compounds. However, the extensive growth of the explored part of chemical space in the XIX century motivated chemists to make efforts to harmonize chemical naming globally. In 1919 International Union of Pure and Applied Chemistry (IUPAC) was founded, and this non-commercial organization still leads the development of chemical nomenclature. IUPAC publishes the Nomenclature of Organic Chemistry, commonly known as the “Blue Book.”^1^ The “Blue Book” provides guidelines on the unambiguous names for chemical compounds.

Nowadays there are several alternative representations for organic structures. For example, Simplified Molecular Input Line Entry System (SMILES) was designed to provide convenience for both human-based and computer-based processing of chemical information. However, IUPAC nomenclature still plays an important role in organic chemistry. The IUPAC notations are obligatory for processing chemicals in many regulated protocols, for example: REACH registration in the EU, patent application submission in many countries, regulatory submission to FDA in the U.S. Most chemical journals require IUPAC names for published organic structures too. Moreover, chemists quite often just prefer to use them. Overall, it is quite probable that the IUPAC nomenclature will be still in use for a while.

In the past, chemists created IUPAC names manually. This process was error-prone because it requires deep knowledge of the nomenclature as well as a high level of attention^2^. It is hard for humans to perform the naming process accurately because it involves a complex algorithm. Moreover, chemists are biased towards trivial names which poses an extra challenge for the proper conversion between different notations. Computers alleviate this problem. Now chemists use software tools for the name generation widely.

The history of names generators begins from the pioneering work of Garfield^3^. However, the first “everyday” software for chemists was created and distributed only at the end of the XX century. Now, there exist several commercial programs for generating IUPAC names: ACD/Labs, ChemDraw, Marvin, IMnova IUPAC Name, etc. Also, there is a framework LexiChem TK that provides an application programming interface (API) for some programming languages^4^. Nevertheless, there is no an open-source tool for the structure-to-name translation. Licensing agreements with the existing solutions, like ChemDraw JS and LexiChem TK, require special permissions for embedding to other platforms.

We note that there is an open-source tool for the name-to-structure translation: OPSIN developed by Daniel Lowe^5^. But, as we mentioned above, there is no one for the inverse problem: structure-to-name conversion.

Recurrent neural networks and Transformer have been successfully used for natural language translation^6,7^. It is worth mentioning that a neural model for direct translation from English to Chinese languages was proposed recently^8^. After our preprint^9^ several studies about the conversion between structural representations and IUPAC names have been published. Rajan et. al. proposed an RNN-based approach for SMILES to IUPAC name conversion^10^. Omote et. al. proposed a multitask Transformer model and byte-pair encoding for the conversion between chemical names and SMILES and InChI strings^11^. An interesting feature of this research was the attempt to convert non-standard chemical names (denoted in PubChem as Synonyms).

We believe that the development costs for a tool for structure-to-name translation “from scratch” are unacceptable in the era of neural networks and artificial intelligence. Instead, we built a Transformer-based neural network that can convert molecules from SMILES representations to IUPAC names and the other way around. In this paper, we describe our solution, discuss its advantages and disadvantages, and show that the Transformer can provide something that resembles human chemical intuition.

Surprisingly, our neural-based solutions achieved a good level of performance that was comparable with the rules-based software. We believe that our approach is suitable for solving the problem of conversions between other technical notations (or other algorithmic challenges) and hope that our findings highlight a new way to resolve issues when the development of a rules-based solution is costly or time-consuming.

## Materials and methods

### Database

Deep learning techniques require large amount of data. PubChem is the largest freely-available collection of chemical compounds with annotations^12^. We used chemical structures and their corresponding IUPAC names from this database. It had 94,726,085 structures in total. The processing and training on the full PubChem database is time-consuming, and about 50M samples seem to be enough for training; so we split the database into two parts and used one half for training and the other one for testing. Structures that can not be processed by RDkit were removed resulting in 47,312,235 structures in the training set and 47,413,850 in the test set.

### IUPAC and SMILES tokenizers

**Figure 1:**
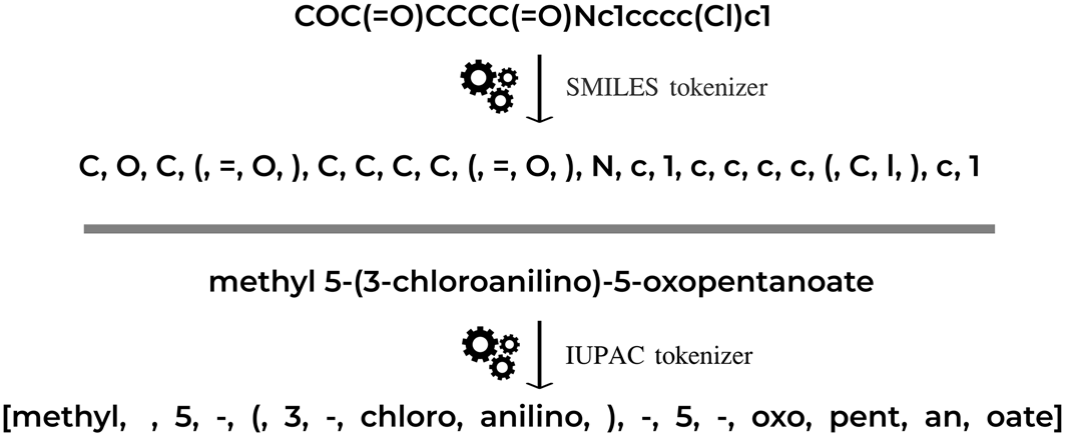
Demonstration of SMILES tokenization (top) and IUPAC names tokenization (bottom).

Tokenization—is a process of partition of a sequence into chunks and demarkation such chunks (tokens). It is a common preprocessing stage for language models. We use a character-based SMILES tokenization and implemented a rule-based IUPAC tokenizer (Fig. 1). Our IUPAC tokenizer was manually designed and curated. We collected all suffixes (-one, -al, -ol, etc.), prefixes (-oxy, -hydroxy, -di, -tri, -tetra, etc.), trivial names (naphthalene, pyrazole, pyran, adamantane, etc.) and special symbols, numbers, stereochemical designations ((, ), [, ], -, N, R(S), E(Z), $\lambda$, etc.). We did not include some tokens in the IUPAC tokenizer because they were very rare or represented trivial names. Also, there is a technical mistake with ”selena” token that makes it impossible to process molecules containing aromatic selenium. We excluded from the training and test sets all molecules that cannot be correctly tokenized. Saying that, we note that our tokenizer was able to correctly process more than 99% of molecules from PubChem.

**Figure 2:**
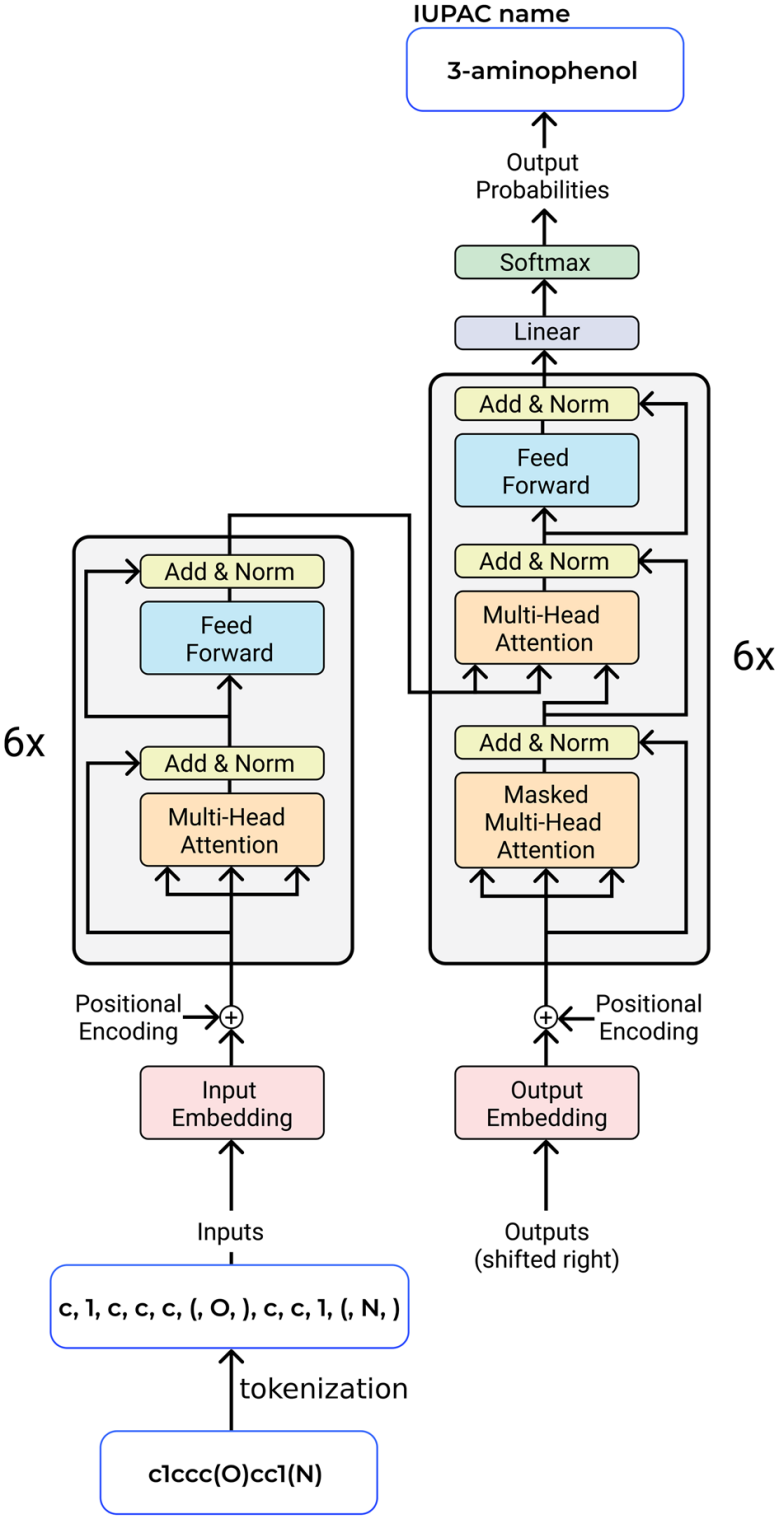
A scheme of *Struct2IUPAC* Transformer. Adopted from^6^.

### Transformer model

Transformer is a modern neural architecture designed by the Google team, mostly to boost the quality of machine translation^6^. Since its origin, Transformer based networks has notably boosted the performance of NLP applications leading to newsworthy GPT models^13^. Transformer has been successfully applied to chemical-related problem: prediction of outcomes of organic reactions^14^, QSAR modelling^15^ and the creation of molecular representations^16^. We used the standard Transformer architecture with 6 encoder and decoder layers, and 8 attention heads. The attention dimension was 512, and the dimension of the feed-forward layer was 2048. We trained two models: *Struct2IUPAC* that converts SMILES strings to IUPAC names and *IUPAC2Srtuct*—that performs reverse conversion. Basically, there is no need for *IUPAC2Srtuct* model because an open-source OPSIN can be successfully used instead. However, studying the reverse conversion performance and following the aesthetic symmetry principle, we created these two models. The schema of our *Struct2IUPAC* model is given in Fig. 2.

### Verification step

**Figure 3:**
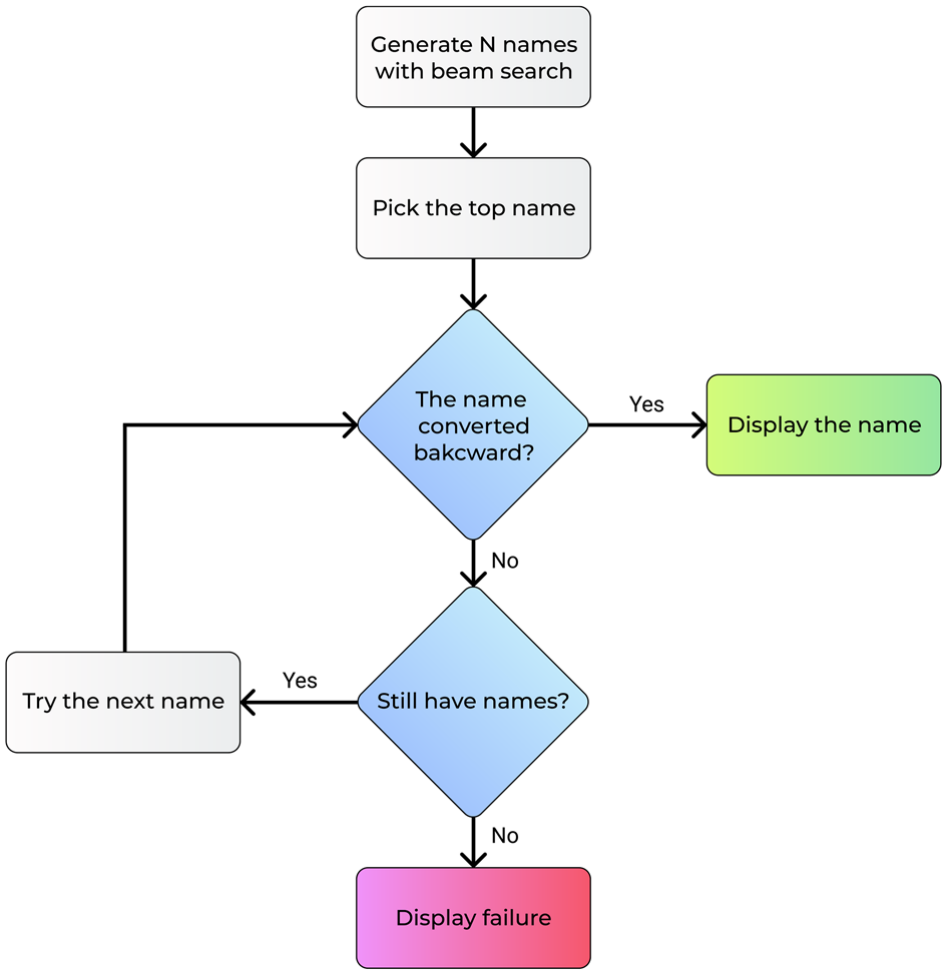
A scheme of Verification step.

Our scheme involves artificial neural networks and its training on data, therefore, the generated solution has a statistical nature with some stochastic components in it. But the generation of a chemical name is a precise task: a name can be either correct or wrong. We believe that the denial of incorrect translation is better than false conversion. Transformer can generate several versions of a sequence using beam search. Using OPSIN we can validate generated chemical names to guarantee that these names correspond to the correct structure. So, we can detect failures of our generator and do not display the wrong name. The flowchart of the verification step is given in Fig. 3.

## Results and discussion

In order to validate the quality of our models we sampled randomly 100,000 molecules from the test set and calculated the percentage of correct predictions with different beam size. Our SMILES to IUPAC names converter, running with verification step, with beam size = 5, achieved 98.9% accuracy (1075 mistakes per 100,000 molecules) on a subset of 100,000 random molecules from the test set. Transformer demonstrates the ability for the precise solution of an algorithmic problem, and this fact raises a new paradigm in software development. Before that there was a consensus opinion that artificial neural networks should not be used if a precise (strict algorithmic) solution is possible. Meanwhile, our approach is built on top of typical neural architecture and requires minimal chemical rules collection (only for tokenizer). The implementation of our system required about one and a half employee months for the whole pipeline. It is hard to estimate the resources required to develop an algorithmic-based generator with competitive performance. Our preliminary estimation about the development of IUPAC names generator “from scratch,” even using the source of OPSIN, would take more than a year by a small team. Anyway, we did not quantify our potential expenses, so we prefer to leave this question to the discretion of the reader. Also, we believe, that our approach can be even more helpful for legacy data. Sometimes there is a lack of documentation for legacy technical notation within the presence of some coincide data. Engineers have to perform ”Rosetta Stone investigations” to make a converter. In our approach, a neural network solves this task saving developers time.

**Figure 4:**
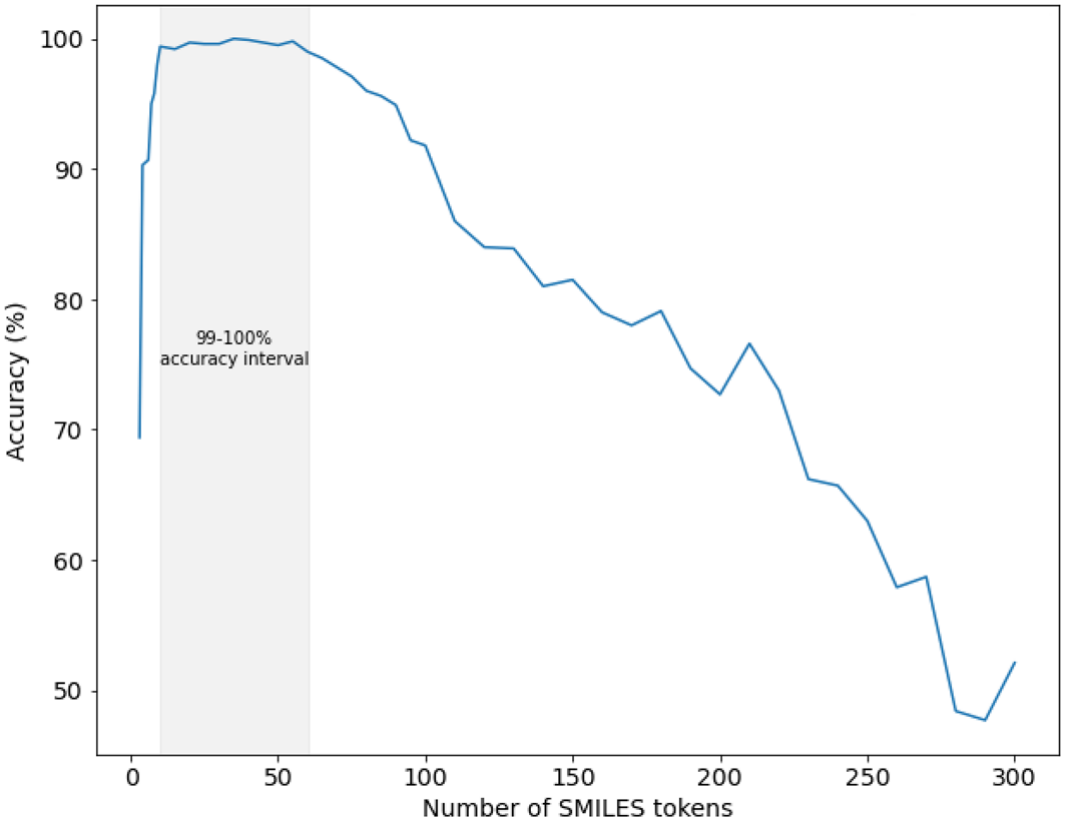
The dependence between model accuracy and the length of SMILES.

**Figure 5:**
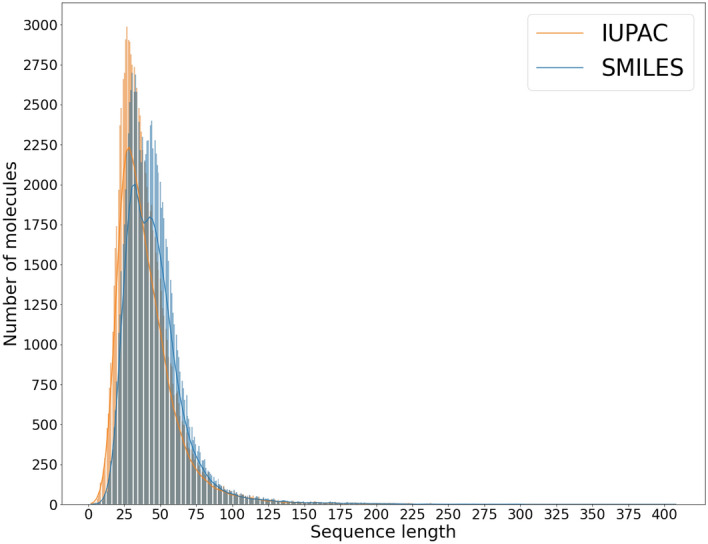
The distribution of the lengths of SMILES and IUPAC on the test set.

Molecules with extra-large number of tokens (oligomers, peptides etc.) are underrepresented in our dataset (see Fig. 5). That can be a possible reason explaining the decline of performance for such molecules. One can also see the apparent decrease of performance for very small molecules. For example, testing the model manually, we found a problematic molecule: methane. A possible explanation could be that the Transformer uses a self-attention mechanism that analyses the correlation between tokens at the input sequence. For an extra-short sequence, it is hard to grasp the relations between tokens; for example, for the extreme example of the methane molecule (one token in SMILES notation), it is just impossible. To estimate the applicability domain of our model we took 1000 examples for each length from 3 to 10 with a step of 1, from 10 to 100 with a step of 5 and 100 to 300 with a step of 10. As a result, we found that our model achieves accuracy close to 100% in the interval from 10 to 60 SMILES tokens. The result of the experiment is given in Fig. 4.

We also showed the distribution of sequence lengths on the test set (Fig. 5). The mean value of the SMILES length is 46,0 tokens and IUPAC length is 40,7 tokens. So the majority of the PubChem molecules is within the applicability domain of our model.

We compared our IUPAC to SMILES Transformer model (*IUPAC2Struct*) with the rules-based tool OPSIN on the test set (Table 1). Our converter achieved 99.1% accuracy (916 mistakes per 100,000 molecules) and OPSIN performed 99.4% (645 mistakes per 100,000 molecules).

**Table 1:** Accuracy (%) of models on the test set of 100k molecules with different beam size.

*Struct2IUPAC*			*IUPAC2Struct*			OPSIN
Beam 1	Beam 3	Beam 5	Beam 1	Beam 3	Beam 5	
96.1	98.2	98.9	96.6	98.6	99.1	99.4

**Figure 6:**
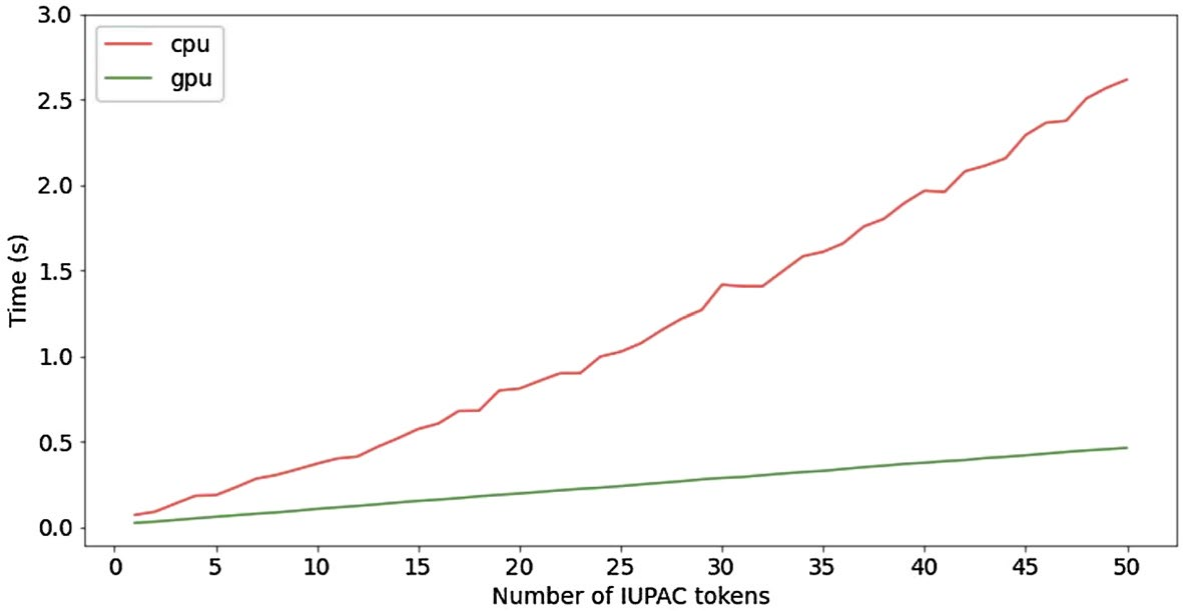
The correlation of mean time and output sequence length.

The Transformer architecture requiures high computational costs. The application of Transformer can be notably slower than an algorithmic-based solutions. To understand the practical applicability of the model in terms of the execution time, we estimated the speed of name generation both on CPU and GPU. We measured the dependence between the number of output IUPAC tokens and the time required for several Transformer runs without beam search and validation feedback loop. The result of the experiment is given in Fig. 6. Transformer consists of two parts: encoder and decoder. Encoder runs only once to read SMILES input, whereas decoder processes each output token. For this reason, only the output sequence length influences the time of execution. One can see that GPU is notably faster than CPU. GPU application requires less than 0.5 seconds even for chemical names with maximal length. This time-frame is acceptable for the practical usage.

Our solution requires signficant computational resources to train the model. The final models has been trained for ten days on a machine with 4 Tesla V100 GPUs and 36 CPUs, with the full load. However, it is still far less expensive than employing human beings for this task.

**Figure 7:**
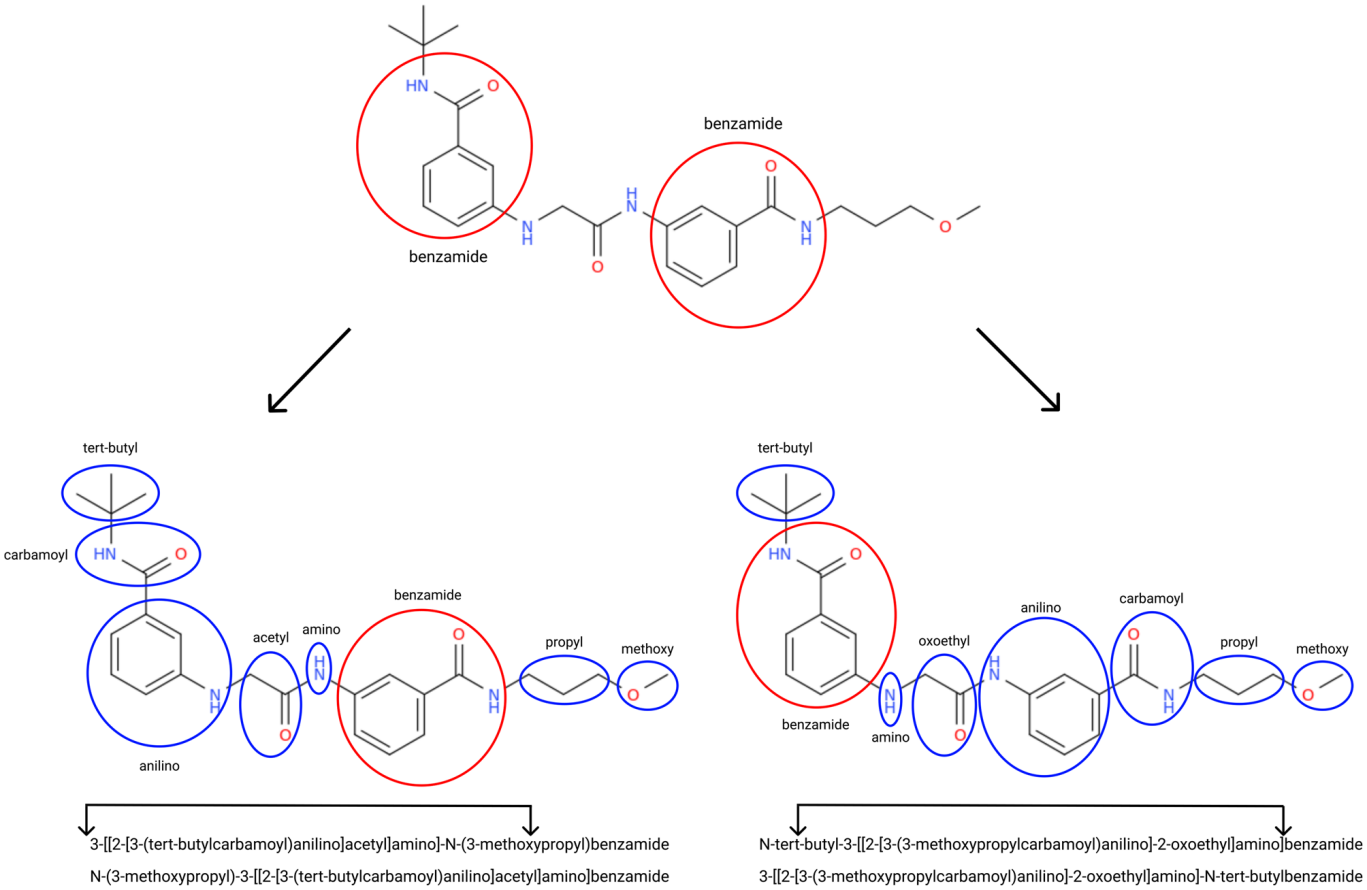
An example of a molecule with four correctly generated IUPAC names.

The most intriguing ability of Transformer is that it can operate with the IUPAC nomenclature in a chemically reasonable way. One can see that the model can infer some chemical knowledge. For example for a molecule on Fig. 7 model generates four chemically correct names (OPSIN converts these IUPAC names to the same structures):

- *3-[[2-[3-(tert-butylcarbamoyl)anilino]acetyl]amino]-N-(3-methoxypropyl)benzamide*
- *N-(3-methoxypropyl)-3-[[2-[3-(tert-butylcarbamoyl)anilino]acetyl]amino]benzamide*
- *N-tert-butyl-3-[[2-[3-(3-methoxypropylcarbamoyl)anilino]-2-oxoethyl]amino]benzamide*
- *3-[[2-[3-(3-methoxypropylcarbamoyl)anilino]-2-oxoethyl]amino]-N-tert-butylbenzamide*

This is due to the fact that the molecule has two parent benzamide groups (in red in Fig. 7). Depending on the choice of the parent group, there are two different ways to name the molecule. Also, for each of the two ways, there are two more ways associated with the substituents enumeration order. As a result, we have four correct IUPAC names for one molecule, and all of these variants are correct.

**Figure 8:**
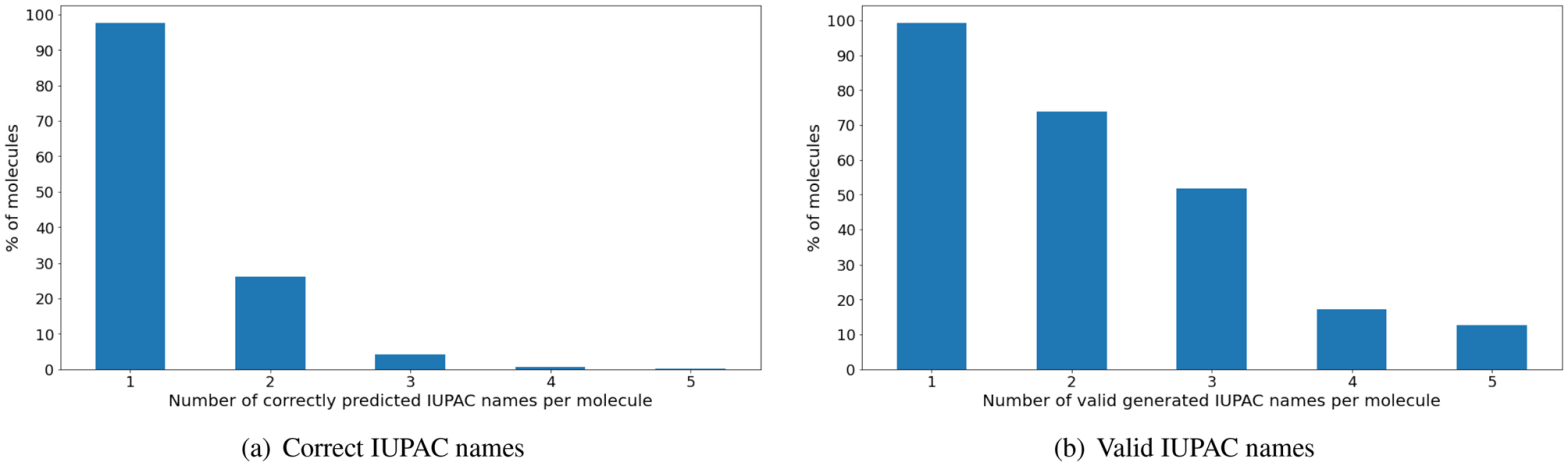
The distribution of the number of names variations using a Transformer’s beam search.

Intrigued by this observation we analyzed the distribution of valid and correct molecules in a batch. We took 10,000 molecules from our 100,000 test subset and calculated: (a) the number of true (correct) IUPAC names (reverse translation by OPSIN leads to the same molecule) (b) the number of chemically valid names (OPSIN can process a molecule, however there is no guarantee that the molecule is the same). These distributions are given in Fig. 8. One can see that Transformer can generate up to two correct names for more than 20% of molecules, and about 1% of molecules can have up to 4 correct IUPAC names. It supports our claims that Transformer does not just memorize common patterns but infers the logic behind IUPAC nomenclature.

**Table 2:** The generated IUPAC names for various tautameric forms of Guanine and Uracil.

Molecule	SMILES	Image	IUPAC names
Guanine	N=c1nc(O)c2[nH]cnc2[nH]1		2-Imino-3,7-dihydropurin-6-ol 2-imino-1,7-dihydropurin-6-ol
	Nc1nc(=O)c2nc[nH]c2[nH]1		2-Amino-3,9-dihydropurin-6-one
	Nc1nc(=O)c2[nH]cnc2[nH]1		2-Amino-3,7-dihydropurin-6-one﻿ 2-amino-6,7-dihydro-3H-purin-6-one 2-amino-3,6-dihydropurin-6-one 2-amino-7H-purin-6-one
	Nc1[nH]c(=O)c2[nH]cnc2n1		2-Amino-1,7-dihydropurin-6-one 2-amino-1,6-dihydropurin-6-one
	Nc1nc(O)c2[nH]cnc2n1		2-Amino-7H-purin-6-ol 2-aminopurin-6-ol
Uracil	O=c1cc[nH]c(=O)[nH]1		1H-Pyrimidine-2,4-dione
	Oc1ccnc(O)n1		Pyrimidine-2,4-diol
	O=c1ccnc(O)[nH]1		2-Hydroxy-1H-pyrimidin-6-one 2-hydroxypyrimidin-6-one
	Oc1cc[nH]c(=O)n1		4-Hydroxy-1H-pyrimidin-2-one 4-hydroxypyrimidin-2-one

An important question is the robustness of our model for various chemical representations: resonance structures, canonical/uncanonical SMILES, etc. The majority of structures are represented as canonical SMILES in unkekulized form in PubChem. To explore the ability of our model to struggle with kekulized SMILES strings, we converted structures to kekulized SMILES by RDKit and calculated the performance on a subset of our test set that contains 10000 molecules. The results of the experiment are given in Table 3. One can see that there is a marginal performance drop; however, the overall quality remains high. The situation is the opposite for augmented (non-canonical SMILES), where we have a tremendous performance drop. The most probable explanation is the lack of non-canonical SMILES in the training set. That is why the model relies on canonical representation. It is worth mentioning that another publication^17^ demonstrates the possibility (or even advisability) of augmented SMILES for Transformer.

Also, we studied the behavior of *Struct2IUPAC* model on compounds with many stereocenters. We took from our test set all compounds with length from 10 to 60 tokens, and calculated an index for each compound: $I=\frac{S}{N}$ where *N* is the number of tokens in a molecule and *S* is the number of stereocenters. We sorted this subset and took the first 10000 compounds. Our test subset was enriched with compounds that have maximal ”stereo-density”—the fraction of stereocenters per token. The results of our model on this subset are given in Table 3. One can see that for the stereo-enriched compounds the performance drops. We have inspected the most common mistakes and saw that the typical errors are in stereo tokens indeed. It is interesting, that in most cases the model tries to vary stereo configurations during the beam search for stereo-enriched compounds. We want to stress that this is the most challenging stereo-compounds from the whole 50M test set and we believe that the demonstrated performance is good regarding the complexity of these compounds.

**Table 3:** The performance of *Struct2IUPAC* model for different validation tasks.

Task	Beam 1 (%)	Beam 3 (%)	Beam 5 (%)
Kekule representation	95.6	97.4	97.7
Augmented SMILES	27.49	34.00	37.16
Stereo-enriched	44.11	61.24	66.52

Another question is the processing of chemical tautomers. If we consider typical types of tautomerism (e.g., keto-enol tautomerism, enamine–imine, etc.), the tautomeric forms are represented by different canonical SMILES and have different IUPAC names. We revealed that our model processes tautomers well, probably because PubChem has a diverse set of tautomeric forms of compounds. The predictions for various tautomers of guanine and uracil are given in Table 2.

It is interesting to track the behavior of Transformer outside the applicability domain. Our observations revealed that the performance of Transformer drops down with large molecules. In the range from 200 to 300 tokens there are two common types of mistakes. The first one is the situation when the model loses the opening or closing squared bracket. It fails the whole structure due to a lexical mistake. That means that the model is undertrained on such an extra-size molecules. This behavior was expected because there were small amount of very large molecules in the training set. The second typical case is losing a part of a large molecule. In this case, Transformer generates a chemically valid molecule, albeit that is shorter than the original. However, Transformer-based models are known for ability to work with thousands-long sequences, and we suppose, that with enough large samples in a dataset, Transformer can achieve good performance on extra large molecules too.

**Figure 9:**
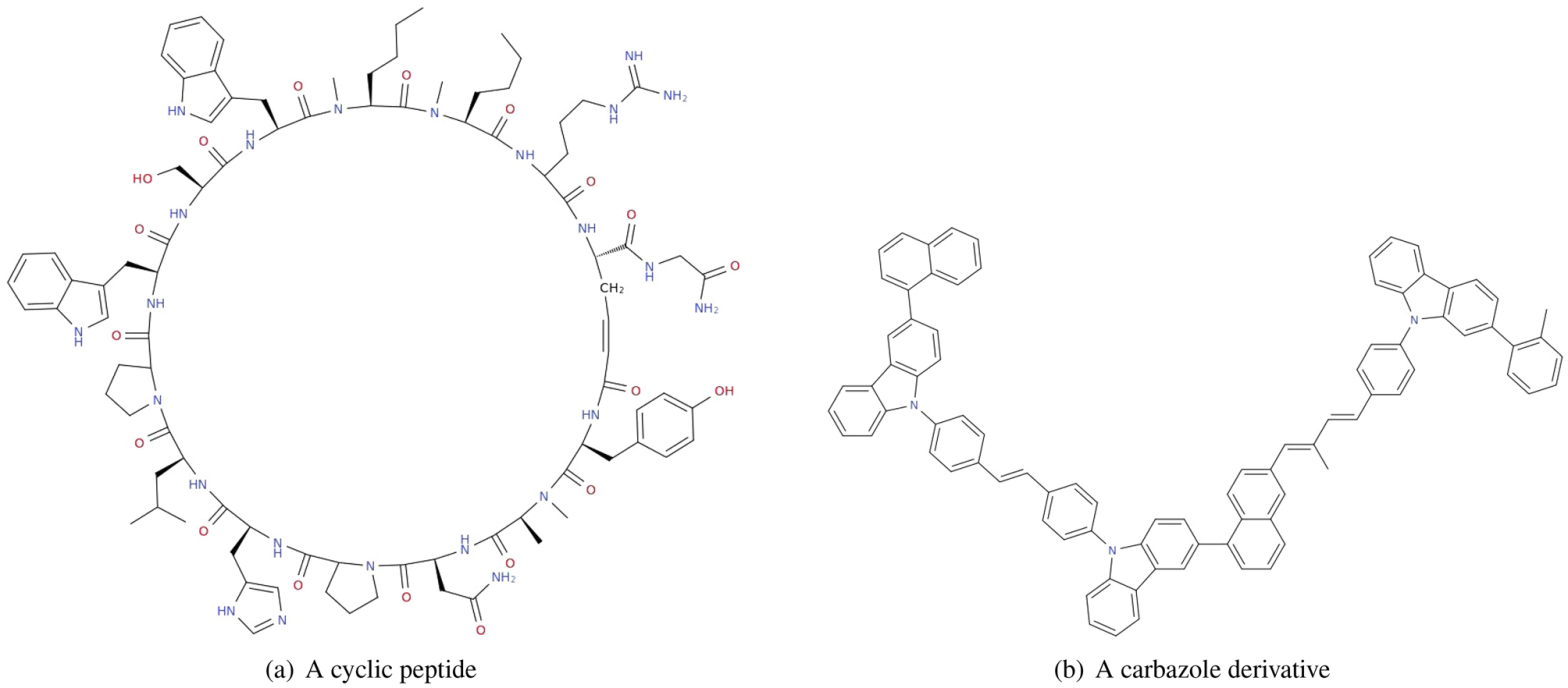
Two examples of challenging molecules for which Transformer generates correct names.

Despite the fact that the accuracy of the model does not exceed 50% on very large molecules, we found the interesting examples of complex molecules for which IUPAC names were correctly generated (Fig. 9).

## Conclusions

In this paper, we propose the Transformer-based solution for generation of IUPAC chemical names using corresponding SMILES notations. This model achieved 98.9% accuracy on the test set from PubChem. Also, the model reached close to 100% accuracy within 10 to 60 tokens length range. Our reverse model (*IUPAC2Struct*) reached accuracy 99.1%, which is comparable to open-source OPSIN software. We demonstrated that the computation time is generally applicable for using this approach in production. We showed that our model operates well within a wide range of molecule size. Our research inspires a new paradigm for software development . We demonstrated that one can replace a complex rule-based solution with modern ”heavy” neural architectures. We believe that neural networks can now solve a wide range of so-called ”exact” problems (problems for which an exact algorithm or solution exists or may exist) with comparable performance. We believe that many groups of researchers and software developers can use and validate this idea for other algorithmic-based challenges.

## Data Availability

The data is located on Zenodo (https://doi.org/10.5281/zenodo.4280814). It contains a subset of 100,000 chemical compounds that were used for testing Transformer, a subset of compounds on which OPSIN fails and compounds on which our *IUPAC2Smiles* model fails. Also, the 100,000 subset is placed on GitHub repository: https://github.com/sergsb/IUPAC2Struct with the *IUPAC2Struct* Transformer code.

## Model availability

Our *Struct2IUPAC* model is available for the community on *Syntelly* platform: https://app.syntelly.com/smiles2iupac—text interface only for testing SMILES to IUPAC model, https://app.syntelly.com/individual—graphical user interface for the prediction of properties of organic compounds and IUPAC names. Transformer and pretrained *IUPAC2Struct* model are available on GitHub: https://github.com/sergsb/IUPAC2Struct.
